# Dynamic Neuro-Cognitive Imagery Improves Mental Imagery Ability, Disease Severity, and Motor and Cognitive Functions in People with Parkinson's Disease

**DOI:** 10.1155/2018/6168507

**Published:** 2018-03-14

**Authors:** Amit Abraham, Ariel Hart, Isaac Andrade, Madeleine E. Hackney

**Affiliations:** ^1^Division of General Medicine and Geriatrics, Department of Medicine, Emory University School of Medicine, Atlanta, GA, USA; ^2^Department of Kinesiology, University of Georgia, Athens, GA, USA; ^3^Emory University College of Arts and Sciences, Atlanta, GA, USA; ^4^Atlanta Department of Veterans Affairs Center for Visual and Neurocognitive Rehabilitation, Atlanta, GA, USA

## Abstract

People with Parkinson's disease (PD) experience kinesthetic deficits, which affect motor and nonmotor functions, including mental imagery. Imagery training is a recommended, yet underresearched, approach in PD rehabilitation. Dynamic Neuro-Cognitive Imagery (DNI™) is a codified method for imagery training. Twenty subjects with idiopathic PD (Hoehn and Yahr stages I–III) were randomly allocated into DNI training (experimental; *n* = 10) or in-home learning and exercise program (control; *n* = 10). Both groups completed at least 16 hours of training within two weeks. DNI training focused on anatomical embodiment and kinesthetic awareness. Imagery abilities, disease severity, and motor and nonmotor functions were assessed pre- and postintervention. The DNI participants improved (*p* < .05) in mental imagery abilities, disease severity, and motor and spatial cognitive functions. Participants also reported improvements in balance, walking, mood, and coordination, and they were more physically active. Both groups strongly agreed they enjoyed their program and were more mentally active. DNI training is a promising rehabilitation method for improving imagery ability, disease severity, and motor and nonmotor functions in people with PD. This training might serve as a complementary PD therapeutic approach. Future studies should explore the effect of DNI on motor learning and control strategies.

## 1. Introduction

Parkinson's disease (PD) affects sensory and cognitive [1–3] as well as motor functions, resulting in impaired proprioception and kinesthesia [3–6]. These deficits manifest as impaired motion sensitivity, joint position sense, spatial cognition, and haptic acuity; altered attention to action; and inaccurate center of gravity [1, 2, 6, 7]. Forty to 63% of people with PD report sensory/perceptual deficits [8, 9] which are more disabling than cardinal PD motor symptoms (e.g., rigidity, tremor, and bradykinesia) during the “off” state (i.e., when anti-Parkinsonian medications are not functioning satisfactorily) [10].

Proprioceptive and kinesthetic deficits are closely linked to [3, 11] and underlie [12] motor deficits in PD. This information composes one's body schema (“body image” [13, 14]) [15], that is, the mental images and proprioceptive representations of the body in relation to the environment [14, 16] that serve as a vital component for perception, action, and motor control [15, 17]. PD-related sensory deficits may facilitate inaccurate body schema [1, 3], further affecting the use and interpretation of proprioceptive information [1, 12, 18] and exacerbating PD motor and cognitive deficits [1, 11, 19]. However, proprioceptive and kinesthetic deficits in PD are often underdiagnosed [20] and have received little attention in PD rehabilitation [12].

Mental imagery (herein referred to as “imagery”) is the cognitive process of creating visual, auditory, or kinesthetic experiences in the mind [21] with or without overt physical execution [22] and is an important tool for cognitive and motor performance [23]. In fact, imagery is a recommended and promising, yet underresearched, tool in PD rehabilitation [24–29], with kinesthetic imagery being particularly recommended [24, 30]. The positive effects of imagery training on people with PD are potentially derived from facilitating conscious motor planning and performance [31]. Imagery relies on [15] and uses [32] proprioceptive and kinesthetic information, including body schema [14, 33–37], thus potentially improving awareness towards body perception and schema [33]. However, proprioceptive and kinesthetic deficits and body schema misperception [13, 16] may affect imagery use in people with PD [19, 25, 38], thus limiting its therapeutic potential for this population. Although PD affects movement speed during imagery [25], imagery ability is generally well preserved in people with PD [25, 39, 40] and was not found to be correlated with the most or least affected side [25, 40]. As a trainable skill driven by internal stimuli [28], imagery ability may be capable of being improved following imagery practice in people with PD [40]. Although not investigated to date, kinesthesia-based imagery interventions may improve imagery ability and use and kinesthetic deficits. This may potentially attenuate cognitive deficits and promote physical performance [30].

Such imagery interventions conform with recommendations in the PD literature [24, 37] because they incorporate sensory information and body awareness for optimizing motor learning [41] and develop correct image properties of actual motor movements [31].

Reports on imagery interventions for people with PD are sparse. A case report describing a 3-month motor imagery (MI; i.e., the cognitive process of mentally rehearsing motor tasks without overt physical movements [42–44]) intervention reported gains in balance, PD motor symptoms, and pain reduction [45]. Furthermore, imagery interventions for people with PD specifically focusing on body schema and kinesthetic could not be found despite being recommended [6, 13].

Reports on imagery interventions embedded within conventional rehabilitation protocols for people with PD are limited and focus on enhancing motor functions through MI [37, 46]. Moreover, the imagery component in these reports is implemented to a limited extent (i.e., 15–20% of the total intervention [37]). In a study assessing the effects of a combined regimen of physical and MI practice (1-hour, biweekly intervention for 12 weeks) with no details regarding the time dedicated to imagery training in a cohort of 23 people with PD [46], the combined MI group showed significant improvements in functional motor task performance times (e.g., standing up and lying down), including the Timed Up and Go (TUG) test (~2.5 sec), the “number of steps required to rotate in a circle,” and UPDRS scores (especially the mentation segment) [46]. Another study assessed the effects of a single session of imagery practice with physical practice versus a single session of physical practice on gait in 20 people with PD. The authors reported that the added imagery practice session did not have a significant effect [47]. Other forms of imagery training, however, have not been explored in PD to date.

Dynamic Neuro-Cognitive Imagery (DNI) (also known as “The Franklin Method” [48–51]) is a codified imagery-based training method for enhancing motor and nonmotor performance. DNI emphasizes correct anatomical and biomechanical embodiment and kinesthetic awareness for mindful and safe movement and function. DNI uses multisensorial, anatomical, and metaphorical imagery techniques [48, 49, 51]. DNI's potential for people with PD lies in compensating for specific PD-related sensory and cognitive mechanisms underlying motor and nonmotor impairments, through enhanced internal imagery-based body representations and sensory information. However, its application to people with PD has not been investigated. Training in DNI has shown gains in biomechanical (i.e., range of motion) (Abraham et al., in preparation) and qualitative (e.g., jump height) aspects [36, 52] of dance performance in university-level dance students, as well as gains in imagery ability and use (Abraham et al., in preparation).

The goals of the study were (1) to assess the feasibility of delivering an intensive, 2-week DNI training for people with PD, (2) to investigate the effects of DNI training versus an in-home learning and exercise program that included frequent staff checkups (herein referred to as “learning/exercise”) on imagery abilities and disease severity and symptoms in a cohort of individuals with mild-moderate PD, and (3) to explore DNI impact on motor, spatial cognitive, and psychological function.

Our hypotheses were as follows: (1) delivering intensive, 2-week DNI training for people with PD will be feasible with high (>80%) retention and adherence rates; (2) participants randomly assigned to DNI training will exhibit greater gains in imagery abilities and disease severity and symptoms compared to participants engaged in the same amount of time in an in-home learning/exercise program over a matched time period; and (3) participants in DNI training will improve more in motor, spatial cognitive, and psychological functions compared to participants in an in-home learning/exercise program.

## 2. Materials and Methods

The study was approved by the Emory University School of Medicine Institutional Review Board. All participants provided written informed consent prior to the beginning of the study.

### 2.1. Participants

Twenty participants with idiopathic PD (Hoehn and Yahr stages I–III) were recruited from the local community through patient support groups, educational events, word of mouth, and the Michael J. Fox Finder website. Inclusion criteria were adults (18 years and more) with a clinical diagnosis of PD based upon established criteria [53] and determined by a board-certified neurologist with specialty training in movement disorders. To clarify, diagnosis of PD required the individual who originally presented with asymmetric symptoms that included at least 3 of the cardinal signs of PD (rigidity, bradykinesia, tremor, and postural instability); must have shown clear symptomatic benefit (e.g., alleviated rigidity, bradykinesia, and tremor) from anti-Parkinsonian medications, for example, levodopa [54]; and must have had unilateral onset of symptoms. For this study, participants also needed to score greater than 17 on the Montreal Cognitive Assessment (MoCA) to be included. Exclusion criteria were any other medical conditions prior to the PD onset potentially causing persistent disability. Participants were aged 40 and older, were stages I–III in the Hoehn and Yahr scale, and could walk 3 meters or more with or without assistance. At the initial assessment, participants were evaluated for general health, self-rated ability to perform activities of daily living, fall risk, age, and education.

### 2.2. Design

Participants were randomly allocated with a computer into either DNI (experimental; *n* = 10) or a learning/exercise (control; *n* = 10) training. Both interventions were conducted simultaneously for 2 weeks and consisted of 5 sessions per week (a total of 10 sessions). Participants were “on,” that is, optimally medicated, during all intervention sessions. Participants were asked to attend a minimum of 4 sessions per week (a total of 8 sessions) and underwent assessments within 1 week before intervention (pretesting) and 2–5 days after the intervention ended (posttesting). Participants and research assistants were blinded to group allocation at pretesting. Participants were asked to maintain their regular medical regimen and all activities during the study.

### 2.3. Experimental Intervention

The DNI intervention was intended to develop participants' imagery skills, kinesthetic and proprioceptive sense, and motor self-awareness. All sessions were delivered in a group by a physical therapist who specialized in imagery training and was also a certified DNI educator. The DNI program was planned by a qualified, experienced instructor (AA), to address PD-specific kinesthetic and proprioceptive deficits, and was developed in line with previous imagery- and PD-related literature [24, 37]. The protocol focused on (1) acquiring imagery skills and techniques (e.g., applying different types, modalities, integration of imagery, and physical movement); (2) correcting anatomical and biomechanical embodiment and kinesthetic and proprioceptive awareness (i.e., understanding the design and function of anatomical structures and identifying their location and motion), focusing on the pelvis, hips, and spine; and (3) using imagery for postural, balance, and coordination enhancement. These contents included, among others, concepts such as dynamic alignment [48] and center of gravity [6] and were all introduced using a broad spectrum of multisensory imagery [48, 49]. An example is given in Table 1. The first session was dedicated to introduction to imagery [34], based on previous literature emphasizing the importance and beneficial effect of introducing imagery as part of an imagery-based intervention in neurorehabilitation [55, 56]. All DNI sessions were conducted at the same time of the day (i.e., mornings) with each session lasting 2 hr (including a break). All DNI sessions followed the same structure: DNI warm-up (15 min), DNI concept introduction and practice part A (35 min), a break (10 min), DNI concept introduction and practice part B (35 min), DNI movement session (20 min), and a DNI cool-down/wrap-up (5 min). The movement session focused on the integration of DNI into movement and exercise and included the use of elastic bands and balls, accompanied by music. Content was practiced individually, in pairs, and in a group. Participants were encouraged to perform according to their ability while trying to “push their boundaries” without risking safety. Able-bodied volunteers who have experience in fall detection and prevention participated in all sessions to assure participants' safety and offered them manual assistance, if needed. Participants were encouraged to practice the DNI techniques and tools at home while performing activities of daily living (ADLs) as well as specific DNI exercises.

### 2.4. Control Intervention

The in-home learning and exercise program, which included staff checkups [57], matched the required time engagement of the DNI group (i.e., 2 hr per day, 5 days per week for two weeks, with a minimum required of 4 sessions per week). Participants were provided with a binder of 8th-grade reading-level lessons related to health and wellness and a 30-minute exercise video, consisting of standing and stepping gross and fine motor exercises that target PD impairments [58]. Participants were instructed to read one lesson per day (estimated time: 1.5 hr) and also make a 30-minute video provided via a secured internet website. All participants had access to the website, but if they had not had such access, we would have provided a DVD for viewing. Lesson topics included the following: research, creativity, exercise, nutrition, infectious diseases, family caregiving, kidney diseases, and health disparities. A research assistant called participants on the telephone 3 times over the 2 weeks (evenly spaced) to confirm compliance and discuss educational content from the lessons. One participant received only 2 calls because they could not be reached for the third phone call. Each call lasted approximately 10 minutes (range: 3 to 20 minutes).

### 2.5. Testing Protocol

The same measurement protocol was administered at pre- and posttesting, using a standardized script with instructions for each task. Participants were assessed with a battery of measures that assessed mental imagery, disease severity and symptoms, and motor and spatial cognitive functions. At posttesting, participants from both groups completed an exit questionnaire to assess their experiences and enjoyment of the intervention [59, 60]. Participants were tested while they were taking medications at a standardized time of day (on state) to reduce potential medication-related fluctuations in performance and came for their visit at a self-determined optimal state.

### 2.6. Cognitive and ADL Status Measures

The *Montreal Cognitive Assessment* (MoCA) is a 30-point test providing a measure of the global status of cognitive impairment through the assessment of a range of executive functions including orientation, memory recall, visuospatial function, attention/concentration, and language. The MoCA achieves high sensitivity and specificity for detecting mild cognitive dysfunction [61] and is valid and reliable in people with PD [62]. If an individual had fewer than 12 years of education, they received an additional point. A score of 27 or greater is considered a normal screen for cognition [61, 63].

The *Composite Physical Function Scale* (CPF) [64] asks 12 questions about an individual's functional ability as related to basic ADLs, intermediate ADLs, and advanced activities. Participants are asked to rate activities as “can do,” “can do with difficulty or assistance,” or “cannot do.” This 24-point scale can provide estimates of risk for loss of function.

### 2.7. Imagery Measures

The *Movement Imagery Questionnaire-Revised Second Version* (MIQ-RS) [65, 66] is a 14-item questionnaire that assesses visual (7 items) and kinesthetic (7 items) imagery ability in people with movement limitations, using gross movements of the trunk and extremities. The examiner first reads the task, participants execute the movement physically and then imagine performing the movement visually or kinesthetically, and then participants score their imagery ease/difficulty. A Visual Analogue Scale (VAS) ranging from 1 (“very hard to see/feel”) to 7 (“very easy to see/feel”) is used with higher scores representing better ability/increased ease.

The *Kinesthetic and Visual Imagery Questionnaire* (KVIQ-20) [67] is a 20-item questionnaire that assesses visual (10 items) and kinesthetic (10 items) imagery ability in people with restricted mobility, using gross and fine motor tasks of the trunk and extremities. The examiner first describes the movement, then demonstrates it, and then the participant is asked to perform the movement, imagine it (using a first-person perspective), and then rate the clarity of the visual imagery or the intensity of the sensations associated with a movement imaged, using a VAS ranging from 1 (“no image/sensation”) to 5 (“image as clear as seeing/as intense as executing the action”) with higher scores reflecting greater imagery ability. The KVIQ-20 was previously used to assess imagery ability in people with PD [25, 31, 40, 68].

The *Vividness of Movement Imagery Questionnaire-Revised Version* (VMIQ-2) [69], previously used in PD [39], is a 36-item questionnaire that assesses the vividness of 3 modes (i.e., external visual, internal visual, and kinesthetic) of movement imagery using 12 actions. VAS ranging from 1 (“perfectly clear and as vivid as normal vision or feel of movement”) to 5 (“no image at all, you only ‘know' that you are thinking of the skill”) is used. Low scores reflect greater imagery ability.

### 2.8. Disease Severity and Psychological Measures

PD-specific measures included the *Movement Disorder Society-Unified Parkinson's Disease Rating Sub-Scales I–IV* (UPDRS I–IV) [70].

Balance confidence was measured with the *Activities-Specific Balance Confidence Scale* (ABC) [71]. The ABC asks 16 questions about an individual's confidence in “not losing his/her balance” in life situations. Participants rate their confidence for each situation on a scale of 0% to 100% confidence. Scores are averaged, and the overall percent confidence was used for analysis.

The *Impact on Participation and Autonomy Scale Questionnaire* (IPA) [72] is a reliable and valid instrument for assessing autonomy and participation in chronic disorders. The IPA measures self-perceived participation in five aspects of life: autonomy indoors, autonomy outdoors, social life, family role, and work/education.

Subjective pain experience was measured with the *Brief Pain Inventory* (BPI; pain severity and interference with daily life) [73], and depression was measured with the *Beck Depression Inventory-II* (BDI-II) [74].

### 2.9. Motor Function Measures

Mobility measures include the *Single and Dual Timed Up and Go* (TUG) test [75] that measures mobility and dual tasking ability [76] with baseline, cognitive (counting backwards by 3 s), and manual (carrying a full glass of water) conditions. Participants rise from a chair, walk 3 meters away, turn, and walk back to the chair and sit down.

*Forward* (Fwd) *Gait Speed* [77] was assessed with a stopwatch. Participants walk 20 feet (~6 meters) and are given a meter of space before and after the 20-foot distance. Gait time and number of steps were measured, allowing for gait speed calculation. Three trials from each condition were averaged.

The *6-Minute Walk Test* (6MWT) measures overall mobility in older people and those with PD [78].

The *30-Second Chair Stand* [79] is a test in which participants rise from a chair to full standing as many times as possible in 30 seconds, without using their hands. The examiner counts aloud the number of repetitions completed.

The *360° Turn Test (Time and Number of Steps)* [80] is a test in which the participant is asked to complete a 360° turn while time to complete and number of steps required to turn are recorded. Right and left directions were tested.

The *Push and Release Test* (PRT) [81] rates the postural response of the participant to a sudden release of the participant pushing backwards on the examiner's hands placed on the participant's back. The VAS scale ranging from 0 (“falls without attempting a step or unable to stand without assistance”) to 4 (“recovers independently with 1 step of normal length and width”) is used.

### 2.10. Cognitive Function Measures

The *Trail Making Test Parts A and B* [82] is a cognitive function test of visual attention, processing speed, executive function, and set switching. In Trail A, a test of visual motor speed and numeric sequencing, the participant connects numbers scattered on the page in ascending order. In Trail B, a test of global frontal lobe dysfunction and executive function, the participant connects numbers and letters on a page in alternating ascending order (i.e., 1-A-2-B-3-C). Participants are required to connect the letters and/or numbers as quickly as possible, without lifting the writing utensil from the paper. They also receive a practice attempt for both Trails A and B. Errors made while completing the task are pointed out immediately so the participant can correct them. Time to complete each trial (up to a maximum of 300 seconds) is recorded. The time difference between Trails A and B (Trail difference = Trail B − Trail A) is considered for analyses.

The *Reverse Corsi Blocks Visuospatial Task* [83] is a test of visuospatial function which requires participants to watch the examiner point to a series of blocks and then repeat the pattern backwards. The examiner begins with two moves and progresses to a maximum of nine moves, with two trials per level. Each level consists of two trials with the same number of moves. At each subsequent level, the number of required moves increases by one move. Participants are given one practice trial of two moves. A participant will advance to the next level if he or she successfully completes at least one of the trials in a level. Once a participant gets both trials of a level incorrect, the task is concluded. The span (total number of moves remembered) and number of trials successfully completed are considered for analyses.

*The Brooks Spatial Memory Task* (BSM) [84] is a test of visuospatial mental imagery in which the participant is asked to visualize a 4 × 4 grid in which the location of numbers 1 through 8 is described. Next, the participant is requested to repeat the numbers' location. Participants practice with three instructions and progress up to 8 instructions. All levels are completed regardless of errors in performance, and percentages correct (out of 50) were used for analysis.

The *Body Position Spatial Task* (BPST) [59] is modelled after the Corsi Blocks task [83], which assesses visuospatial short-term working memory. Whereas with Corsi Blocks the examiner points to a sequence of blocks in a particular spatial pattern coded by numbers, in BPST, the examiner demonstrates (verbally and visually) a sequenced pattern of steps to the side, forward, and turning (in place). The participant then repeats the pattern exactly. The examiner begins with two moves and progresses to a maximum of nine moves. At each subsequent level, the number of required moves increases by one move, with two trials per level. Participants are given one practice trial of two moves. Participants advance to the next level if they correctly complete at least one of the trials in a level. Once a participant misses both trials of a level, the task is concluded. The span (number of moves remembered) and number of trials performed correctly are used for analyses. Participants are allowed to use their assistive device (i.e., a cane or a walker) if they use it habitually.

### 2.11. Participants' Satisfaction Measures

An exit questionnaire [59, 60] was administered to all participants after the intervention for assessing whether participants enjoyed the intervention or would continue and whether they noted improvements in aspects of well-being. A VAS ranging from 1 (“strongly agree”) to 5 (“strongly disagree”) was used.

### 2.12. Statistical Analysis

The last observation was carried forward for participants who did not complete a minimum of 8 of the 10 offered sessions for their group assignment (DNI or learning/exercise) before posttesting (*n* = 2). Data were analyzed using SPSS (Version 19.0, IBM Corp., Armonk, NY). Descriptive statistics and two-way [group (DNI, learning/exercise) × time (pre, post)] mixed-design analysis of variance (ANOVA) were used to assess the effect of the interventions. Two-tailed hypotheses were used with a *p* value of .05 or less regarded as significant. Effect sizes (*η*_p_^2^) and confidence intervals (95% CI) were also calculated. For the 360° Turn Test, data were analyzed for the right side only after conducting paired-sample *t*-tests which yielded nonsignificant differences (*p* > .05) between sides for both time and number of steps.

## 3. Results

Participants' demographics are displayed in Table 2. At pretesting, the groups had normal cognition (as assessed by MoCA), there were more males, about half of them had experienced falls in the previous year or used an assistive device, they were at low risk for losing function (as measured by the CPF), and they had mild-moderate PD. The groups differed slightly in cognitive function as assessed by MoCA (Table 2), but both groups were within the normal range for cognition (i.e., >26 points). There were no significant differences between groups in imagery ability, as measured by MIQ-RS, KVIQ-20, and VMIQ-2 (not shown) nor between visual and kinesthetic imagery abilities at pretesting (Table 3).

Delivering both the DNI and learning/exercise interventions was feasible with high adherence and compliance. Two participants did not complete the intervention and posttesting: One DNI participant fell at home while skateboarding recreationally, resulting in back pain that prevented him from attending, and one learning/exercise participant could not complete assignments because of Parkinsonian complications.

Compliance for the DNI group was 100%. All participants in learning/exercise read the information and discussed with staff (verified by staff), but 4 out of 9 learning/exercise participants did not complete the exercise video 8 times as requested. All participants used the video at least twice.

### 3.1. Outcome Measures

Results for all outcome measures are detailed in Table 4. There was a group × time interaction in the mental imagery measures. The DNI group improved more than the learning/exercise group did in all mental imagery measures except for the kinesthetic MIQ-RS and kinesthetic VMIQ-2.

There were significant group × time interactions in the UPDRS-III, the TUG-manual, time and number of steps to turn 360°, reactive postural control, and BPST span. The DNI group improved more than the learning/exercise group did.

There was a significant group × time interaction in the IPA score. The learning/exercise group improved more.

Both groups strongly agreed that they enjoyed their program and strongly agreed that they were more mentally active and would continue the program if possible. DNI participants agreed that they noted improvements in balance, walking, mood, and coordination, and they were more physically active. Median and interquartile values are reported for exit questionnaire responses in Table 5.

## 4. Discussion

This study represents one of the first efforts to examine the effects of imagery training on imagery ability, disease severity, motor, and nonmotor functions in people with mild-to-moderate PD. The DNI intervention provided participants with imagery information based on correct body biomechanics and encouraged them to use this knowledge for increasing self-awareness and improving motor performance. This is different from other approaches to imagery, in which participants' existing motor experiences serve as the foundation for the imagery training [37]. Further, the DNI intervention included imagery contents only (unlike previous reports using combined imagery and conventional therapies), thus resulting in a high volume of imagery training (100 min per session) in comparison to previous reports (e.g., 15–20 minutes [46] and 20 minutes [37]).

Delivering intensive, 2-week DNI training was feasible with high adherence and compliance rates, thus confirming our first hypothesis. Participants in the DNI group enjoyed the training, attended sessions beyond the minimum required, and commented that the training was very useful for them in improving their ADLs. The DNI group benefitted significantly more from the training versus the learning/exercise group and exhibited greater improvements in measures of imagery ability, disease severity, and motor and nonmotor functions.

The lack of significant differences between visual and kinesthetic imagery ability found at pretesting agrees with previous findings [40] but contradicts other findings suggesting visual imagery to be better than kinesthetic imagery in PD [25, 68]. Kinesthetic and visual imagery trainings may be equally relevant and potentially beneficial for people with PD. Future studies, however, should compare between the two modalities in terms of their effectiveness in this population.

MIQ-RS and KVIQ-20 scores in the current study could not be compared with previous reports of people with PD due to significant differences in delivery protocol [68] or VAS scales [25, 31, 40]. There is therefore a need for homogenous delivery protocols in this population. VMIQ-2 scores in the current study (23.30 ± 12.68, 30.35 ± 13.86, and 32.05 ± 13.55 for external-visual, internal-visual, and kinesthetic abilities, resp.) were higher than previous published values (27.5 ± 7.4 and 28.3 ± 6 for internal-visual and kinesthetic) in a group of 15 people with PD [39]. In another study, a mean score of 24.4 ± 7.1 was reported, with no specifications regarding the imagery modality [85].

The DNI group improved significantly in all three modalities of imagery ability (i.e., external-visual, internal-visual, and kinesthetic) following the intervention, whereas the learning/exercise group did not exhibit these improvements, which match, and may be explained by, the multi-imagery perspectives (i.e., 1st- and 3rd-person) and multisensorial imagery approach (i.e., visual, auditory, and kinesthetic) used by DNI. This finding supports the notion that imagery ability can be enhanced following imagery training in people with PD and suggests that imagery performance (e.g., vividness) in people with PD can improve not only with actual visual cues (i.e., a target on a screen) [31] but also with imagery training.

Previous literature suggests that people with PD are less likely to use a “first-person” strategy for imagery (i.e., kinesthetic or internal-visual), which involves internal-visual or kinesthetic information [29], possibly reflecting kinesthetic and proprioceptive deficits [1, 4, 7] and altered body schema [6, 16, 86] associated with PD. Therefore, the improvements in kinesthetic and internal-visual and imagery abilities noticed following the DNI training may suggest an enhanced ability to access kinesthetic, internal information, thus potentially more likely to use a first-person imagery modality. Such improvements in kinesthetic and internal-visual imagery ability may lead to better somatosensory integration and body schema [14, 33], as well as reduced external (e.g., auditory and visual) cue or feedback dependency, frequently observed in PD [18]. Being more available and not dependent on space and time constraints, relying on internally guided, kinesthetic stimuli and cues, has potential for enhancing independence and decreased reliance on externally provided cues in ADLs for people with PD, thus improving the quality of life [31, 41]. Future studies should investigate the effect of DNI training on the ability of people with PD to spontaneously generate and use internally generated cues [87]. Such insights, once demonstrated, could be translated into clinical guidelines and embedded within PD rehabilitation protocols, as previously suggested with imagery [26, 27].

This study is the first, to our knowledge, to demonstrate significant improvement in UPDRS-III, that is, motor symptoms, following imagery training. A previous study reported a significant improvement in the mental subscale (UPDRS-I) following a combined physical and motor imagery training [46]. Gaps in knowledge prohibit explaining the relationships between different types of imagery training or contents and the effects on aspects of disease severity, as measured by the UPDRS.

The DNI group improved significantly more than the learning/exercise group did in selected measures of motor and cognitive functions, including TUG-manual and 360° turns. These improvements are especially notable because the DNI intervention focused on anatomical and biomechanical embodiment and kinesthetic-proprioceptive imagery and not on the training of specific functions/tasks to be measured as outcome measures (e.g., TUG-manual and 6MW), as is the case in previous reports [37, 46]. As such, the current findings might suggest changes in motor control and planning strategies, involved in such functions, following relevant DNI contents, such as imagery for embodying the center of mass and central axis. These aspects were not within the scope of this study and should be looked at in future works.

The mechanisms of effect of DNI are not fully revealed to date and should be examined in light of previous literature highlighting brain strategies and compensatory mechanisms involved with imagery in PD [88]. Specifically, such investigations should take into account previous reports suggesting that performance of an imagery task (i.e., mental rotation of body parts; as measured by reaction time and brain activity) in people with PD is affected by various factors, such as visual information (e.g., image orientation), the affected/nonaffected limb, and the presence of tremor [88, 89]. Moreover, it was concluded that people with PD used first-person, kinesthetic imagery to solve the imagery task [89].

The improvements noted in the DNI group in motor and nonmotor functions could be explained, in part, by the following: (1) imagery plays an important role in motor and nonmotor functions [23], and gains in imagery ability could contribute to enhanced motor and nonmotor capabilities; (2) correcting (and enhancing) body posture and biomechanics is important for proper motor performance [90, 91]; and (3) providing additional sensory information could optimize motor learning in people with PD [41]. In addition, the imagery-cognitive strategies used in DNI could potentially serve to bypass the basal ganglia-supplementary motor area circuit [46] and contribute to the establishment of a more clear and accurate body schema [14].

This study has limitations: (1) it evaluated only a small sample, (2) differences existed between groups on the MoCA and imagery experience, (3) the intervention was only two weeks long, and (4) there was a lack of follow-up testing to evaluate the retention effect. In addition, four out of the 9 participants in the learning/exercise group reported that they did not complete the exercise video 8 times as requested, which resulted in a reduced volume of the control intervention for these participants. All of these limitations have affected the results in unknown ways; therefore, the current findings should be interpreted cautiously. Future studies should investigate the effects of DNI training with larger samples and longer interventions with long-term follow-up measurements.

Future research should also explore the potential for beneficial effects of DNI in people with PD in other circumstances, for example, during the off state [40] and when combined with other training approaches, for example, physical therapy group exercise.

## 5. Conclusion

This study provides preliminary evidence for the clinical application and effectiveness of imagery training in PD. The demonstrated gains in imagery ability and motor and nonmotor functions in people with PD following DNI training further support the incorporation of imagery training in PD rehabilitation. Research into imagery and motor control and planning in PD is warranted.

## Figures and Tables

**Figure 1:**
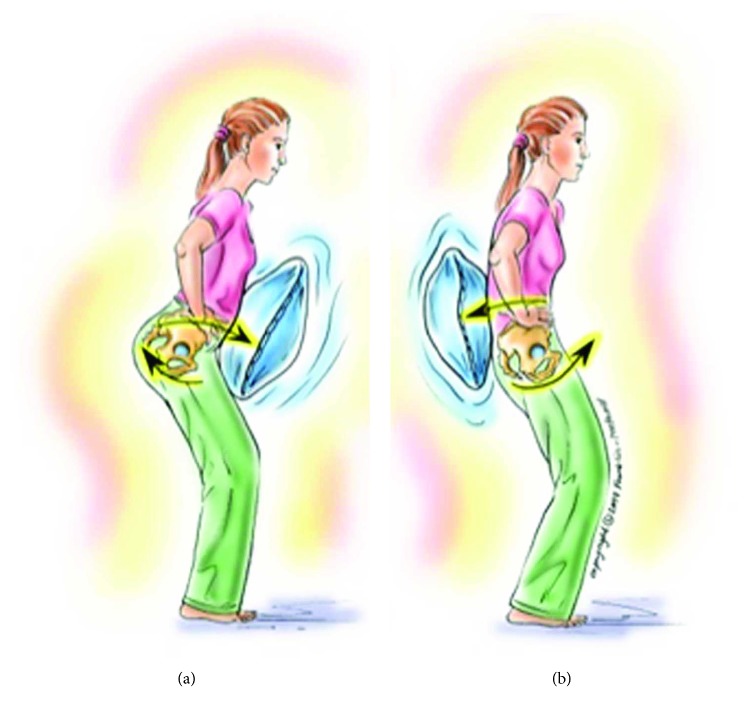
DNI “pushing the pelvis into a big pillow.”

**Figure 2:**
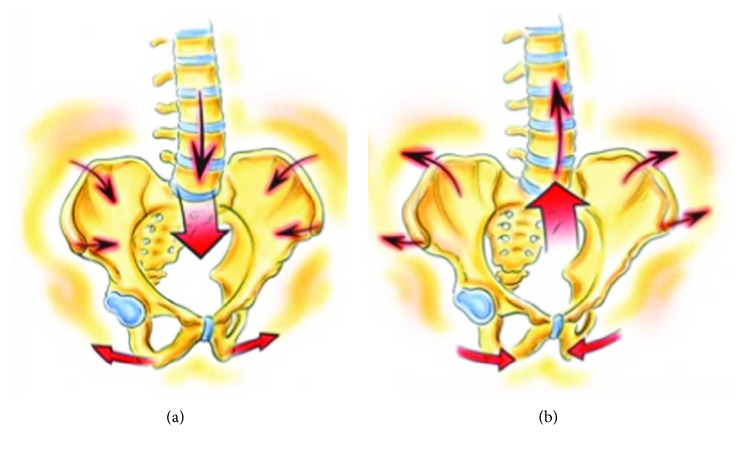
DNI pelvic parts moving in APT (a) and PPT (b).

**Figure 3:**
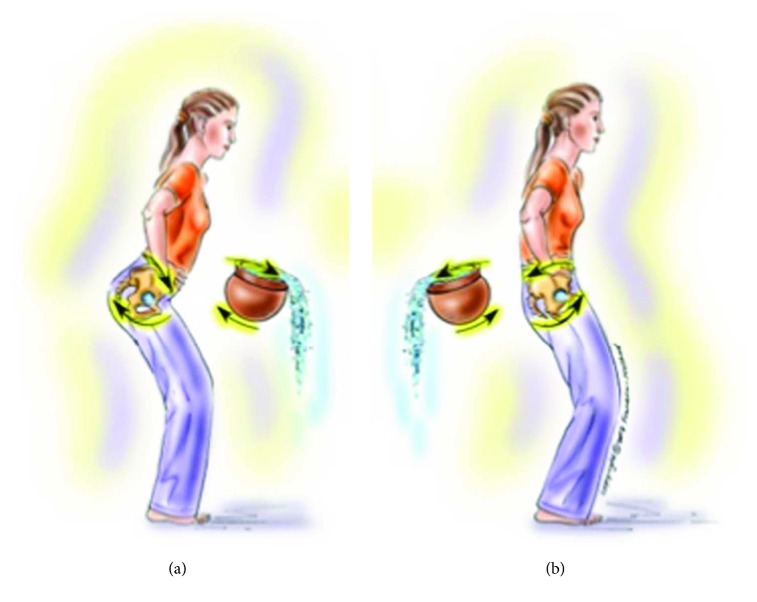
DNI “pelvis as a bowl and water pouring out of it to the front (in APT (a)) and to the back (in PPT (b)).”

**Table 1:** Multisensorial DNI for enhancing anterior and posterior pelvic tilt.

	DNI exercises
Anatomical embodiment	Self-touch: touching the iliac crests and innominates and imagining/feeling them moving throughout pelvic tilting
Kinesthetic	Pushing the low back (in PPT) and abdomen (in APT) into a big pillow (Figure 1)
Visual-cognitive	Watching a pelvic model and visualizing the different parts (i.e., two innominates and sacrum) moving in the desired manner (Figure 2)
Auditory-cognitive	Saying out loud: “pelvis is tilting forward” (for APT) and “pelvis is tilting backward” (for PPT)
Metaphorical	The pelvis is a bowl pouring water anteriorly (in APT) and posteriorly (in PPT) (Figure 3)
Auditory	Listening to the sound of pouring water (using 2 cups filled with water)

Note: APT = anterior pelvic tilt; PPT = posterior pelvic tilt. All drawings are presented with permission from Mr. E. Franklin.

**Table 2:** Baseline participants' demographics (*M*, SD).

	DNI (*n* = 10) *M* (SD)	Learning/exercise (*n* = 10) *M* (SD)	*p* ^a^
Sex	1 woman, 9 men	3 women, 7 men	.58^b^
Age (years)	66.4 (12.5)	65.1 (7.5)	.78
UPDRS Motor Subscale III	38.4 (13.8)	32.1 (12.2)	.29
Hoehn and Yahr stage (median (first, third quartiles))	2.0 (1.8, 2.5)	2.0 (2.0, 2.5)	.80
Duration of PD (years)	6.1 (3.8)	8.5 (4.5)	.21
MoCA (/30)	28.3 (1.4)	26.6 (2.0)	.04^∗^
CPF (/24)	19.9 (4.6)	20.3 (3.71)	.83
Education (years)	16.2 (2.2)	16.4 (2.0)	.83
Number of comorbidities	3.4 (1.7)	2.6 (1.7)	.32
Number of prescription medications	5.7 (3.4)	3.1 (3.1)	.09^c^
Use of assistive device (yes/no)	3/7	4/6	1.00^b^
History of ≥1 falls in the past year (yes/no)	6/4	4/6	.65^b^
Previous experience with imagery (yes/no)	4/6	1/9	.30^b^

Note: values are mean (SD), unless otherwise noted. PD = Parkinson's disease; UPDRS = Unified Parkinson's Disease Rating Sub-Scale; MoCA = Montreal Cognitive Assessment; CPF = Composite Physical Function Scale. ^a^Independent *t*-tests' compared groups. ^b^Fisher's exact test. ^c^Equal variance not assumed. ^∗^*p* < .05.

**Table 3:** Visual and kinesthetic imagery abilities at pretesting.

	Visual *M* (SD)	Kinesthetic *M* (SD)	*p* ^a^
MIQ-RS (/7)	4.86 (1.64)	4.68 (1.63)	.72
KVIQ-20 (/5)	3.19 (1.04)	2.86 (.93)	.28
VMIQ-2^†^ (12–70)	External: 29.30 (12.68) Internal: 30.35 (13.86)	32.05 (13.55)	.47^b^

^a^Paired-sample *t-*tests' compared categories. ^b^Paired-sample *t-*tests' compared internal-visual and kinesthetic categories. ^**†**^Lower values represent better scores.

**Table 4:** Outcome measures at pre- and posttesting.

	DNI *M* (SD) [range] {95% CI}	Learning/exercise *M* (SD) [range] {95% CI}	*F*_(1,18)_^!^	*p*	*η*_p_^2^
Mental imagery					
*MIQ-RS (/7)*					
Visual					
Pre	4.98 (1.56) [1.86–6.71] {3.86–6.10}	4.74 (1.79) [1.00–6.43] {3.62–5.86}	5.84	.02^∗^	.245
Post	5.85 (.79) [4.71–7.00] {4.89–6.81}	4.31 (1.88) [1.00–6.57] {3.35–5.27}			
Kinesthetic					
Pre	5.07 (1.43) [2.29–6.57] {3.99–6.15}	4.30 (1.79) [1.00–6.29] {3.22–5.38}	1.69	.20	.086
Post	5.47 (1.48) [2.29–7.00] {4.31–6.62}	4.05 (1.96) [1.00–6.57] {2.90–5.21}			
Total					
Pre	5.02 (1.14) [3.36–6.64] {4.21–5.83}	4.52 (1.29) [2.50–6.36] {3.71–5.33}	5.30	.03^∗^	.228
Post	5.66 (.96) [4.14–7.00] {4.85–6.47}	4.18 (1.43) [2.21–6.57] {3.37–4.99}			
*KVIQ-20 (/5)*					
Visual					
Pre	3.29 (1.22) [1.30–4.80] {2.58–3.99}	3.10 (.88) [1.50–4.00] {2.39–3.80}	6.12	.02^∗^	.254
Post	3.95 (.63) [3.00–4.90] {3.31–4.58}	2.67 (1.18) [1.00–4.20] {2.03–3.30}			
Kinesthetic					
Pre	3.07 (1.10) [1.00–4.70] {2.44–3.69}	2.66 (.72) [1.30–3.50] {2.03–3.28}	6.58	.01^∗^	.268
Post	3.65 (1.18) [1.00–5.00] {2.94–4.35}	2.39 (.92) [1.00–4.10] {1.68–3.09}			
Total					
Pre	3.18 (.89) [2.00–4.75] {2.69–3.66}	2.88 (.51) [2.10–3.75] {2.39–3.36}	9.62	.00^∗∗^	.348
Post	3.80 (.80) [2.50–4.90] {3.27–4.32}	2.53 (.77) [1.25–3.45] {2.00–3.05}			
*VMIQ-2^†^ (/12–70)*					
External visual					
Pre	26.10 (11.68) [12.00–47.00] {17.73–34.46}	32.50 (13.42) [20.00–60.00] {24.13–40.86}	6.70	.01^∗^	.271
Post	21.20 (6.64) [12.00–31.00] {15.10–27.29}	36.40 (11.13) [19.00–60.00] {30.30–42.49}			
Internal visual					
Pre	27.70 (15.09) [12.00–56.00] {18.42–36.97}	33.00 (12.73) [19.00–60.00] {23.72–42.27}	5.79	.02^∗^	.244
Post	20.00 (7.39) [12.00–35.00] {12.66–27.33}	36.30 (13.76) [17.00–60.00] {28.96–43.63}			
Kinesthetic					
Pre	29.40 (14.53) [12.00–53.00] {20.33–38.46}	34.70 (12.69) [14.00–60.00] {25.63–43.76}	1.96	.17	.098
Post	22.10 (11.44) [12.00–48.00] {14.02–30.17}	34.70 (12.83) [15.00–60.00] {26.62–42.77}			

Disease severity and psychological					
UPDRS-I^†^					
Pre	11.90 (5.19) {7.67–16.12}	15.00 (7.34) {10.77–19.22}	.00	1.00	.000
Post	10.40 (5.58) {6.15–14.64}	13.50 (7.10) {9.25–17.74}			
UPDRS-II^†^					
Pre	16.50 (5.87) {12.82–20.17}	18.20 (5.18) {14.52–21.87}	.01	.90	.001
Post	14.10 (6.17) {10.33–17.86}	15.60 (5.12) {11.83–19.36}			
UPDRS-III^†^					
Pre	38.40 (13.87) {29.70–47.09}	32.10 (12.24) {23.40–40.79}	4.08	.05^∗^	.185
Post	31.60 (13.85) {22.96–40.23}	31.20 (12.06) {22.56–39.83}			
UPDRS-IV^†^					
Pre	3.50 (4.11) {0.89–6.10}	4.60 (3.71) {1.99–7.20}	.02	.87	.001
Post	4.50 (5.03) {1.84–7.15}	5.40 (2.54) {2.74–8.05}			
ABC (%)					
Pre	78.20 (18.31) {67.14–89.26}	73.20 (14.79) {62.14–84.26}	.00	.94	.000
Post	74.23 (18.02) {67.61–90.27}	74.23 (18.02) {62.90–85.57}			
IPA^†^					
Pre	32.60 (21.80) {14.96–50.23}	43.50 (30.54) {25.86–61.13}	5.20	.03^∗^	.224
Post	35.00 (22.28) {20.03–49.96}	36.20 (22.75) {21.23–51.16}			
BPI-severity^†^					
Pre	2.82 (2.25) {1.22–4.42}	3.03 (2.56) {1.23–4.82}	.95	.34	.056
Post	2.55 (2.25) {1.05–4.04}	3.28 (2.20) {1.60–4.95}			
BPI-interference^†^					
Pre	2.44 (1.79) {.86–4.01}	2.21 (2.90) {.45–3.97}	2.48	.13	.134
Post	1.41 (1.24) {.40–2.42}	2.14 (1.79) {1.01–3.27}			
BDI-II^†^					
Pre	12.90 (8.41) {6.93–18.86}	14.50 (9.51) {8.53–20.46}	.75	.39	.040
Post	9.20 (5.82) {4.73–13.66}	12.40 (7.50) {7.93–16.86}			

Motor function					
Fwd gait^†^ (meters/sec)					
Pre	1.08 (.18) {.95–1.21}	1.06 (.20) {.93–1.19}	.00	.92	.001
Post	1.09 (.15) {.98–1.20}	1.07 (.16) {.96–1.17}			
6MWT (meters)					
Pre	410.52 (127.05) {348.89–472.14}	393.34 (32.61) {331.72–454.96}	2.46	.13	.120
Post	447.32 (84.22) {399.53–495.11}	370.05 (57.06) {322.26–417.85}			
30-Second Chair Stand (reps)					
Pre	14.20 (6.52) {10.60–17.80}	11.10 (4.01) {7.50–14.70}	1.49	.23	.077
Post	16.00 (5.61) {12.69–19.30}	11.40 (4.24) {8.09–14.70}			
Mini-BEST (/28)					
Pre	22.50 (4.37) {19.68–25.31}	21.80 (4.10) {18.68–25.31}	1.60	.22	.082
Post	23.30 (3.30) {20.86–25.73}	21.20 (3.99) {18.76–23.63}			
TUG^†^ (sec)					
Pre	9.00 (2.18) {7.61–10.40}	9.85 (2.02) {8.45–11.25}	2.21	.15	.110
Post	8.40 (1.85) {7.00–9.80}	10.26 (2.32) {8.86–11.66}			
TUG-cognitive^†^ (sec)					
Pre	13.72 (8.07) {9.57–17.87}	12.76 (3.57) {8.61–16.91}	3.07	.09	.146
Post	11.14 (3.21) {6.65–15.63}	15.61 (9.01) {11.12–20.11}			
TUG-manual^†^ (sec)					
Pre	13.61 (5.44) {10.75–16.46}	12.69 (2.71) {9.83–15.55}	4.43	.05^∗^	.198
Post	11.16 (2.76) {9.28–13.03}	12.99 (2.87) {11.11–14.87}			
360° Turn Test (steps)^†^					
Pre	10.20 (5.57) {7.43–12.96}	6.90 (1.91) {4.13–9.66}	6.63	.01^∗∗^	.269
Post	9.00 (2.53) {6.26–11.73}	9.70 (5.22) {6.96–12.43}			
360° Turn Test (sec)^†^					
Pre	4.23 (2.98) {2.75–5.70}	3.12 (.95) {1.64–4.59}	7.58	.01^∗∗^	.296
Post	3.38 (1.46) {1.96–4.80}	4.69 (2.64) {3.27–6.11}			
PRT (/4)					
Pre	2.80 (1.31) {2.07–3.52}	2.70 (.82) {1.97–3.42}	5.68	.02^∗^	.240
Post	3.40 (.84) {2.82–3.97}	2.10 (.87) {1.52–2.67}			

Cognitive function					
Trail A^†^ (sec)					
Pre	27.46 (10.33) {20.64–34.28}	30.44 (10.19) {23.62–37.26}	1.24	.27	.065
Post	28.19 (7.03) {23.51–32.88}	27.68 (7.06) {23.00–32.37}			
Trail B^†^ (sec)					
Pre	78.26 (52.49) {32.03–124.49}	95.79 (83.23) {49.56–142.02}	.01	.90	.001
Post	68.29 (29.35) {28.34–108.23}	87.52 (79.80) {47.58–127.47}			
Trails B-A^†^ (sec)					
Pre	50.79 (45.11) {8.70–92.89}	65.34 (77.42) {23.24–107.44}	.18	.66	.010
Post	40.09 (25.35) {2.91–77.27}	59.84 (74.97) {22.66–97.02}			
Corsi-trials					
Pre	6.30 (1.82) {4.91–7.68}	6.70 (2.31) {5.31–8.08}	1.87	.18	.094
Post	7.30 (1.49) {5.97–8.62}	6.80 (2.39) {5.47–8.12}			
Corsi-span					
Pre	4.70 (1.15) {3.98–5.41}	4.90 (.99) {4.18–5.61}	1.05	.31	.055
Post	5.30 (1.05) {4.47–6.13}	5.00 (1.41) {4.17–5.83}			
BSM					
Pre	72.40 (14.07) {58.92–85.87}	66.40 (24.99) {52.92–79.87}	.19	.66	.011
Post	77.60 (14.56) {65.53–89.66}	68.20 (21.15) {56.13–80.26}			
BPST-trials					
Pre	4.10 (1.52) {3.14–5.05}	4.40 (1.34) {3.44–5.35}	.48	.49	.026
Post	4.70 (1.56) {3.48–5.91}	4.60 (2.06) {3.38–5.81}			
BPST-span					
Pre	3.40 (.69) {2.60–4.19}	4.20 (1.54) {3.40–4.99}	6.68	.01^∗∗^	.271
Post	3.90 (.99) {3.19–4.60}	3.80 (1.13) {3.09–4.50}			

Note: values represent group × time interactions. MIQ-RS = Movement Imagery Questionnaire-Revised Second Version; KVIQ = Kinesthetic and Visual Imagery Questionnaire; VMIQ = Vividness of Movement Imagery Questionnaire-Revised Version; UPDRS = Unified Parkinson's Disease Rating Sub-Scales; ABC = Activities-Specific Balance Confidence Scale; IPA = Impact on Participation and Autonomy Scale Questionnaire; BPI = Brief Pain Inventory; BDI = Beck Depression Inventory; 6MWT = 6-Minute Walk Test; TUG = Timed Up and Go; FAB = Fullerton Advanced Balance Scale; DGI = Dynamic Gait Index; PRT = Push and Release Test; BSM = Brooks Spatial Memory Task; BPST = Body Position Spatial Task. ^**!**^Differences were calculated using mixed-design ANOVA. ^∗^*p* < .05. ^∗∗^*p* < .01. ^**†**^Lower values represent better scores.

**Table 5:** Participants' satisfaction from intervention.^†^

Question	DNI (median (1st, 3rd quartiles))	Learning/exercise (median (1st, 3rd quartiles))
(1) Did you enjoy?	1.00 (1.00, 1.00)	1.00 (1.00, 2.00)
(2) Balance improved	2.00 (1.00, 3.00)	3.00 (3.00, 3.00)
(3) Walking improved	2.00 (1.50, 2.00)	3.00 (2.50, 3.50)
(4) Mood improved	2.00 (1.00, 3.00)	3.00 (2.00, 3.50)
(5) Coordination improved	2.00 (2.00, 3.00)	3.00 (3.00, 4.00)
(6) Strength improved	3.00 (1.00, 3.00)	3.00 (3.00, 3.00)
(7) Endurance improved	3.00 (3.00, 3.00)	3.00 (3.00, 3.50)
(8) I would continue	1.00 (1.00, 1.00)	2.00 (1.00, 3.50)
(9) More physically active	2.00 (1.00, 2.50)	3.00 (2.00, 3.50)
(10) More mentally active	1.00 (1.00, 2.00)	2.00 (1.50, 3.50)

^†^Lower values represent better scores.
